# A Semi-Quantum Secret-Sharing Protocol with a High Channel Capacity

**DOI:** 10.3390/e25050742

**Published:** 2023-04-30

**Authors:** Yuan Tian, Genqing Bian, Jinyong Chang, Ying Tang, Jian Li, Chongqiang Ye

**Affiliations:** 1 College of Information and Control Engineering, Xi’an University of Architecture and Technology, Xi’an 710055, China; 2 School of Cyberspace Security, Beijing University of Posts and Telecommunications, Beijing 100876, China

**Keywords:** quantum cryptography, semi-quantum secret sharing, hyper-entangled states, degree of freedom, eavesdropping detection

## Abstract

Semi-quantum cryptography communication stipulates that the quantum user has complete quantum capabilities, and the classical user has limited quantum capabilities, only being able to perform the following operations: (1) measuring and preparing qubits with a Z basis and (2) returning qubits without any processing. Secret sharing requires participants to work together to obtain complete secret information, which ensures the security of the secret information. In the semi-quantum secret sharing (SQSS) protocol, the quantum user Alice divides the secret information into two parts and gives them to two classical participants. Only when they cooperate can they obtain Alice’s original secret information. The quantum states with multiple degrees of freedom (DoFs) are defined as hyper-entangled states. Based on the hyper-entangled single-photon states, an efficient SQSS protocol is proposed. The security analysis proves that the protocol can effectively resist well-known attacks. Compared with the existing protocols, this protocol uses hyper-entangled states to expand the channel capacity. The transmission efficiency is 100% higher than that of single-degree-of-freedom (DoF) single-photon states, providing an innovative scheme for the design of the SQSS protocol in quantum communication networks. This research also provides a theoretical basis for the practical application of semi-quantum cryptography communication.

## 1. Introduction

The core idea of secret sharing is that the secret holder appropriately divides a piece of complete secret information into several parts, leaving these parts of the secret information to different participants for safekeeping [1]. A single participant or part cannot obtain adequate secret information. Only when all participants cooperate can complete secret information be recovered. Secret sharing achieves decentralized management of secret information and plays a role in reducing the risk of eavesdropping and tolerating some attacks and errors [2]. Moreover, the secret sharing protocol is vital in practical applications such as key agreement, secure multi-party computing, and voting systems [3,4]. Based on the excellent characteristics of quantum mechanics, such as non-cloning, quantum cryptography theoretically provides a scheme for designing protocols with unconditional security [5,6]. It can also detect whether there is eavesdropping in the communication process. The concept of quantum cryptography was proposed in 1984 [7], when Bennett and Brassard designed the first quantum key distribution (QKD) protocol using non-orthogonal quantum states and the uncertainty principle. The protocol only needs to use a single particle state, which is easy to implement, and its security has been strictly proven. As a popular cryptography technology, quantum cryptography has received extensive attention and in-depth research [8,9,10]. The quantum secret sharing (QSS) protocol is an essential branch of the quantum cryptography protocol, which can be used to share confidential information between multiple participants. In 1999, Hilllery et al. studied the QSS protocol based on the Greenberger–Horne–Zeiligner (GHZ) states for the first time [11] and proposed a protocol for sharing classical secret and quantum secret information. Later, many scholars joined the research of QSS [12,13,14].

However, people’s research on quantum information technology is still in its infancy. Quantum communication, quantum computing, and other technologies are more complex and challenging to apply in current science and technology, work, and life [15,16]. Moreover, quantum devices have high prices and complex operations, and quantum state preparation, storage, and transmission are also extremely complex. The proposal of semi-quantum cryptography effectively alleviates the bottleneck in the development of quantum cryptography. This is relatively easier to realize while ensuring security. The semi-quantum key distribution (SQKD) protocol allows one party to have the full quantum capability, while the other party’s quantum capability is limited to achieve secure communication between a quantum user and a “classical user” [17]. Semi-quantum cryptography has attracted the attention of many scholars and is one of the emerging research hotspots in quantum cryptography [18,19,20,21,22,23,24,25,26]. In semi-quantum cryptography, some users can only hold simple quantum devices, saving the high cost of purchasing quantum devices. Some users can prepare quantum states without the need to prepare quantum states or by only using the computational basis (Z basis), which reduces the complexity of preparing quantum states. Especially in case of equipment failure in the quantum cryptography communication process, users can choose to switch the quantum cryptography communication mode to a semi-quantum cryptography communication mode to ensure the completion of the whole communication process.

In 2010, using the concept of “semi-quantum”, Li et al. proposed two SQSS protocols for the first time [27]. Subsequently, this attracted a large number of scholars to study SQSS. In 2013, Li et al. considered it difficult to prepare entangled states in practice and designed a more realistic SQSS protocol based on the product states [28]. Xie et al. used two entangled states to encode messages for SQSS [29] and realized the direct sharing of secret information in 2015. Based on the Bell states, efficient SQSS was proposed by Yin et al. in 2017 [30]. The next year, Li et al. proposed an SQSS protocol which enables the sharing of secret information without the participants making any measurement operations [31]. In 2019, Xiang et al. presented a new SQSS scheme based on multi-level quantum systems which can share a large amount of information of the third and fourth levels with the same security [32]. Tsai et al., based on the W state, proposed a three-party SQSS protocol [33]. In 2021, Tian et al. constructed a new SQSS protocol which can share specific secret information and is more efficient than similar protocols [34]. An SQSS protocol for high-dimensional quantum systems based on product states was proposed in the context of the qualifications of Hu et al. in 2022 [35]. It is easy to find that the existing SQSS protocols focus on using the characteristics of different qubits to design different protocols, and all SQSS protocol quantum carriers are only in one DoF. Therefore, this paper, based on hyper-entangled states, implements a new SQSS protocol for the first time: the transmission particles in polarization and spatial mode DoFs.

As a carrier of quantum information, photons can not only become entangled in a single DoF, such as polarization, path, or spatial mode, but also realize entanglement in multiple DoFs at the same time, which is called hyper-entanglement [36,37]. Compared with a single DoF, photons in multiple DoFs have many advantages: they can achieve more effective state measurements, build an asymmetric optical quantum network, improve the channel capacity of the quantum network, and also contribute to the physical realization of quantum purification and quantum computation. In recent years, research on hyper-entangled states has become a hot topic in the field of quantum information, and many significant advances have been made, such as the preparation of hyper-entangled states and multi-DoF teleportation based on hyper-entangled states.

In this paper, we employ the unique advantages of hyper-entangled states to construct a novel SQSS protocol based on hyper-entangled single-photon states. The hyper-entangled state improves the communication capacity, and the amount of information that the proposed protocol can share in one communication process is twice that of the single-DoF protocols. In addition, we analyze three attack strategies—intercept-resend attack, measure-resend attack, and entangle-measure attack—and prove that the proposed protocol can effectively resist well-known attacks. Table 1 lists all explanations of acronyms and symbols used in the paper.

The structure of this paper is as follows: Section 2 describes the proposed SQSS protocol, Section 3 analyzes the security of three attacks, and Section 4 discusses and concludes this paper.

## 2. Protocol

Generally, the N hyper-entangled single-photon states in polarization and spatial mode DoFs can be represented by

(1) $$\begin{array}{c} {|\Pi \rangle }_{PS}=|\delta_{P}\rangle_{AB\ldots Z}⊗{|\delta_{S}\rangle }_{AB\ldots Z}, \end{array}$$

where the subscript AB…Z denotes the N photons, *P* denotes the polarization DoF, and *S* denotes the spatial-mode DoF. There are two non-orthogonal measure bases in the polarization DoF: the basis $Z_{P}=\{|H\rangle ,|V\rangle \}$ and $X_{P}=\{|R\rangle ,|A\rangle \}$. Here, we have

(2) $$\begin{array}{c} {|R\rangle }_{P}=\frac{|H\rangle +|V\rangle }{\sqrt{2}}, \end{array}$$

(3) $$\begin{array}{c} {|A\rangle }_{P}=\frac{|H\rangle -|V\rangle }{\sqrt{2}}, \end{array}$$

where |H〉 and |V〉 indicate the horizontal and vertical polarizations. There are two non-orthogonal measure bases in the spatial mode DoF: the basis $Z_{S}=\{|x_{1}\rangle ,|x_{2}\rangle \}$ and $X_{S}=\{|a\rangle ,|b\rangle \}$. Here, we have

(4) $$\begin{array}{c} {|a\rangle }_{S}=\frac{|x_{1}\rangle +|x_{2}\rangle }{\sqrt{2}}, \end{array}$$

(5) $$\begin{array}{c} {|b\rangle }_{S}=\frac{|x_{1}\rangle -|x_{2}\rangle }{\sqrt{2}}, \end{array}$$

where $|x_{1}\rangle$ and $|x_{2}\rangle$ indicate the upper and the lower spatial modes, respectively.

There are N single photons in the polarization DoF, which can be described as

(6) $$\begin{array}{c} |\delta_{P}\rangle_{AB\ldots Z}={\left(\frac{|H\rangle \pm |V\rangle }{\sqrt{2}}\right)}_{A}{\left(\frac{|H\rangle \pm |V\rangle }{\sqrt{2}}\right)}_{B}\ldots {\left(\frac{|H\rangle \pm |V\rangle }{\sqrt{2}}\right)}_{Z}. \end{array}$$

There are N single photons in the spatial mode DoF, which can be described as

(7) $$\begin{array}{c} |\delta_{S}\rangle_{AB\ldots Z}={\left(\frac{|x_{1}\rangle \pm |x_{2}\rangle }{\sqrt{2}}\right)}_{A}{\left(\frac{|x_{1}\rangle \pm |x_{2}\rangle }{\sqrt{2}}\right)}_{B}\ldots {\left(\frac{|x_{1}\rangle \pm |x_{2}\rangle }{\sqrt{2}}\right)}_{Z}. \end{array}$$

We set N=2, and the hyper-entangled single-photon states are

(8) $$\begin{array}{cc} \; & {|\Gamma \rangle }_{PS}={|\gamma \rangle }_{P}⊗{|\gamma \rangle }_{S}\\ \; & ={|R\rangle }_{P}{|R\rangle }_{P}⊗{|a\rangle }_{S}{|a\rangle }_{S}\\ \; & =\frac{|H\rangle +|V\rangle }{\sqrt{2}}\frac{|H\rangle +|V\rangle }{\sqrt{2}}⊗\frac{|x_{1}\rangle +|x_{2}\rangle }{\sqrt{2}}\frac{|x_{1}\rangle +|x_{2}\rangle }{\sqrt{2}}. \end{array}$$

The three-party SQSS protocol based on hyper-entangled single-photon states is presented in detail. Alice, as the holder of the secret information, plans to share the secret with two classical participants: Bob and Charlie. Here, Alice is a quantum user who has full quantum capabilities. She can generate and measure qubits with any basis. Bob (Charlie) is a classical user whose quantum capabilities are restricted. He can only use the Z basis to generate and measure qubits. The processing of the proposed protocol is as follows:

**Step 1: Carrier preparation and transmission.** Alice generates *n* two-qubit product hyper-entangled states ${|\Gamma \rangle }_{PS}$:

(9) $$\begin{array}{cc} \; & {|\Gamma \rangle }_{PS}={|\gamma \rangle }_{P}^{BC}⊗{|\gamma \rangle }_{S}^{BC}\\ \; & ={|R\rangle }_{P}^{B}{|R\rangle }_{P}^{C}⊗{|a\rangle }_{S}^{B}{|a\rangle }_{S}^{C}\\ \; & =\frac{{|H\rangle }^{B}+{|V\rangle }^{B}}{\sqrt{2}}\frac{{|H\rangle }^{C}+{|V\rangle }^{C}}{\sqrt{2}}⊗\frac{|x_{1}\rangle^{B}+{|x_{2}\rangle }^{B}}{\sqrt{2}}\frac{|x_{1}\rangle^{C}+{|x_{2}\rangle }^{C}}{\sqrt{2}}, \end{array}$$

where *B* and *C* denote the system of the two participants: Bob and Charlie, respectively. Alice forms a quantum sequence *S* using ${|\Gamma \rangle }_{PS}$:

(10) $$\begin{array}{cc} \; & S={\{{[|R\rangle }_{P}^{}⊗|a\rangle }_{S}^{}{\left(B\right),|R\rangle }_{P}^{}⊗{|a\rangle }_{S}^{}{\left(C\right)]}_{1},{[|R\rangle }_{P}^{}⊗{|a\rangle }_{S}^{}{\left(B\right),|R\rangle }_{P}^{}⊗{|a\rangle }_{S}^{}{\left(C\right)]}_{2},\\ \; & \ldots ,{[|R\rangle }_{P}^{}⊗{|a\rangle }_{S}^{}{\left(B\right),|R\rangle }_{P}^{}⊗{|a\rangle }_{S}^{}\left(C\right){]}_{n}\}. \end{array}$$

Then, Alice divides the sequence *S* into sequences $S_{B}$ and $S_{C}$:

(11) $$\begin{array}{c} S_{B}={\{{[|R\rangle }_{P}^{}⊗|a\rangle }_{S}^{}{\left(B\right)]}_{1},{[|R\rangle }_{P}^{}⊗{|a\rangle }_{S}^{}{\left(B\right)]}_{2},\ldots ,{[|R\rangle }_{P}^{}⊗{|a\rangle }_{S}^{}\left(B\right){]}_{n}\}, \end{array}$$

(12) $$\begin{array}{c} S_{C}={\{{[|R\rangle }_{P}^{}⊗|a\rangle }_{S}^{}{\left(C\right)]}_{1},{[|R\rangle }_{P}^{}⊗{|a\rangle }_{S}^{}{\left(C\right)]}_{2},\ldots ,{[|R\rangle }_{P}^{}⊗{|a\rangle }_{S}^{}\left(C\right){]}_{n}\}, \end{array}$$

Alice then sends $S_{B}$ to Bob and $S_{C}$ to Charlie.

**Step 2: Bob and Charlie’s operations.** When Bob (Charlie) receives the sequence $S_{B}$ ($S_{C}$), Bob (Charlie) randomly chooses the SIFT or CTRL operation. The SIFT operation means the participant measures the qubits with the Z basis, prepares fresh qubits with the same measurements with the Z basis, and sends them to Alice. The CTRL operation means the participant reflects the qubits to Alice without disturbance.

**Step 3: Alice, Bob, and Charlie’s publication.** For the last qubits arriving from Bob and Charlie, Alice broadcasts that the sequences $S_{B}$ and $S_{C}$ have been received. After acknowledgment, Bob and Charlie announce the certain operation, SIFT or CTRL, which has been chosen for each qubit.

**Step 4: Alice’s operations.** According to Bob’s (Charlie’s) choice, Alice divides the corresponding qubits into the following four cases. The four cases as illustrated in Table 2 and depend on the different operations which Bob and Charlie performed. In case 1, Bob and Charlie chose SIFT, and Alice measured the qubits *B* and *C* with $Z_{P}⊗Z_{S}$. In case 2, Bob chose SIFT and Charlie chose CTRL, while Alice measured the qubits *B* with $Z_{P}⊗Z_{S}$ and the qubits *C* with $X_{P}⊗X_{S}$. In case 3, Bob chose CTRL and Charlie chose SIFT, while Alice measured the qubits *B* with $X_{P}⊗X_{S}$ and the qubits *C* with $Z_{P}⊗Z_{S}$. In case 4, Bob and Charlie chose CTRL, and Alice measured the qubits *B* and *C* with $X_{P}⊗X_{S}$. Case 1 was used for generating the raw keys and checking for eavesdropping, while cases 2, 3, and 4 were used for checking for eavesdropping.

**Step 5: First eavesdropping detection.** Alice conducted eavesdropping detection in cases 2, 3, abd 4. Alice measured the measured qubits with $Z_{P}⊗Z_{S}$ and measured the reflected qubits with $X_{P}⊗X_{S}$. For example, in case 2, Alice would measure the qubits *C* with $X_{P}⊗X_{S}$, which were reflected by Charlie. If there was no eavesdropper, then the measurement results should be same as in the initial state which she prepared. Alice would measure the qubits *B* with $Z_{P}⊗Z_{S}$, which were measured by Bob, and she would inform Bob to publish his measurement results. If there was no eavesdropper, then the measurement results should be the same between Alice and Bob. Similarly, Alice would have the same operations for eavesdropping as in case 3. In case 4, Alice would measure the qubits *B* and *C* with $X_{P}⊗X_{S}$, and the measurement results should be same as the initial state which was prepared by her; otherwise, there was an eavesdropper present. If the error rate is higher than the predefined threshold values, then Alice terminates the protocol. The details of the security analysis will be provided in the next section.

**Step 6: Second eavesdropping detection.** Alice randomly selects a few qubits which belong to case 1 to be TEST bits and announces the states and corresponding positions. Bob and Charlie compare the states with their qubits. Alice and Bob (Charlie) should have the same measurement results. If the error rates exceed the threshold values, then the protocol aborts.

**Step 7: Secret sharing.** The remaining qubits in case 1 are INFO bits. Because of the hyper-entangled states in polarization and spatial mode DoFs, one participant’s operations on one qubit position can share two keys. Secret information 1 and 2 are encoded by Alice as given in Table 3. $K_{A}$, $K_{B}$, and $K_{C}$ are the results measured by Alice, Bob, and Charlie in case 1, respectively. Presume that $K_{A}$ is the secret bits, and Alice encodes $K_{A}$ as

(13) $$\begin{array}{c} K_{A}\equiv K_{B}⊕K_{C}. \end{array}$$

Only Bob and Charlie’s colleagues can obtain the secret information.

## 3. Security Analysis

Suppose that an eavesdropper wants to obtain Alice’s secret information through illegal means. He will take specific means to eavesdrop on the participants’ keys. Generally, when analyzing the security of the SQSS protocol, a dishonest participant (Bob or Charlie) causes more serious harm than external eavesdroppers because they have already obtained one part of the secret information and only need to steal the secret information of another participant. They can obtain Alice’s secret information alone. Therefore, the security analysis of the proposed protocol mainly focuses on malicious participants. Assuming that Bob is a malicious participant, he uses the following attack strategies to steal Charlie’s keys and finally obtains Alice’s secret information.

**Measure-resend attack.** To obtain Charlie’s secret keys, Bob uses the measure-resend attack strategy. When Alice sends $S_{C}$ to Charlie, Bob first intercepts the sequence, measures the qubits using the $Z_{P}⊗Z_{S}$ basis, and stores the measurement results. Then, Bob prepares a new sequence $S_{E}$ with $Z_{P}⊗Z_{S}$, which is the same state as the previous sequence measured by him. If Alice and Charlie do not detect Bob’s attack, then Bob will obtain the corresponding Charlie secret keys according to his measurement results. Unfortunately, Bob’s eavesdropping can be detected through eavesdropping detection in cases 2 and 4 under step 5 because Bob cannot distinguish which operation Charlie will choose for the specific qubits. When Bob measured ${[|R\rangle }_{P}^{}⊗{|a\rangle }_{S}^{}{\left(C\right)]}_{i}$, where Charlie chose the SIFT operation on ${[|R\rangle }_{P}^{}⊗{|a\rangle }_{S}^{}{\left(C\right)]}_{i}$, he could successfully obtain Charlie’s secret keys and combine his secret keys to calculate Alice’s secret information. Although such behavior will not introduce any errors, once Charlie selects the CTRL operation, Alice will have a chance of half to detect the errors introduced by Bob when performing eavesdropping detection. Specifically, with Alice using the $X_{P}⊗X_{S}$ basis to measure the qubits prepared by Bob, the measurement results may occur as one of four results: ${\{{[|R\rangle }_{P}^{}⊗|a\rangle }_{S}^{}{\left(C\right)]}_{i},{[|R\rangle }_{P}^{}⊗{|b\rangle }_{S}^{}{\left(C\right)]}_{i},{[|A\rangle }_{P}^{}⊗{|a\rangle }_{S}^{}{\left(C\right)]}_{i}$, and ${[|A\rangle }_{P}^{}⊗{|b\rangle }_{S}^{}\left(C\right){]}_{i}\}$. Thus, the error rate introduced by Bob can be calculated as $r_{1}=\frac{1}{2}\times \frac{3}{4}=\frac{3}{8}$, and he can eavesdrop the probability of $\frac{5}{8}$. The detection probability for the presented SQSS protocol is $p_{1}=1-{\left(\frac{5}{8}\right)}^{t}$. If *t* is large enough, then the detection probability will be toward one.

**Intercept-resend attack.** Bob employs the intercept-resend attack strategy to steal Charlie’s secret keys. First, Bob intercepts the sequence $S_{C}$ and sends a fake sequence $S_{E}^{\prime }$ with $Z_{P}⊗Z_{S}$ or $X_{P}⊗X_{S}$ to Charlie. Then, Charlie sends back $S_{E}^{\prime \prime }$ to Alice. Bob intercepts $S_{E}^{\prime \prime }$ and sends $S_{C}$ to Alice. Unfortunately, Bob will inevitably be detected. When Charlie chose the CTRL operation, there was no error introduced by Bob. However, there was a possibility of a half that Charlie chose the SIFT operation. In the case where Charlie chose SIFT, Bob would be detected in cases 1 and 3 under step 5. Specifically, Alice used the $Z_{P}⊗Z_{S}$ basis to measure the qubits returned by Bob. The measurement results may occur as one of four results: ${\{{[|x_{1}\rangle }_{P}^{}⊗|x_{1}\rangle }_{S}^{}\left(C\right){]}_{i},{[|x_{1}\rangle }_{P}^{}⊗|x_{2}\rangle_{S}^{}\left(C\right){]}_{i},{[|x_{2}\rangle }_{P}^{}⊗|x_{1}{\rangle_{S}^{}\left(C\right)]}_{i}$, and ${[|x_{2}\rangle }_{P}^{}⊗|x_{2}\rangle_{S}^{}\left(C\right){]}_{i}\}$. Thus, the error rate introduced by Bob can be calculated as $r_{2}=\frac{1}{2}\times \frac{3}{4}=\frac{3}{8}$. He can eavesdrop with a probability of $\frac{5}{8}$. When *t* is large enough, the detection probability of $p_{2}=1-{\left(\frac{5}{8}\right)}^{t}$ will approximately be one for the presented SQSS protocol.

**Entangle-measure attack.** Bob’s most general attack is the measure-entangle attack, which is composed of two unitary operations: ${\bar{U}}_{E}$ attacking qubits when Alice sends information to Charlie and ${\bar{U}}_{F}$ attacking qubits when Charlie sends information to Alice.

**Theorem** **1**. *Suppose that Bob uses an attack (${\bar{U}}_{E}$,${\bar{U}}_{F}$) on the qubits from Alice to Charlie and from Charlie back to Alice. Furthermore, the final probes should be independent of Charlie’s measurement results to not induce any error. Hence, Bob cannot obtain any information on the secret keys.*

**Proof.**  Before Bob’s attack, the qubits are ${|R\rangle }_{P}^{}⊗{|a\rangle }_{S}^{}$, and the ancillary qubit is |e〉. After ${\bar{U}}_{E}$, the state evolves into

(14) $$\begin{array}{c} {\bar{U}}_{E}{(|R\rangle }_{P}^{}⊗{|a\rangle }_{S}^{})|e\rangle =|Hx_{1}\rangle |e_{Hx_{1}}\rangle +|Hx_{2}\rangle |e_{Hx_{2}}\rangle +|Vx_{1}\rangle |e_{Vx_{1}}\rangle +|Vx_{2}\rangle |e_{Vx_{2}}\rangle , \end{array}$$

where $|e_{Hx_{1}}\rangle$, $|e_{Hx_{2}}\rangle$, $|e_{Vx_{1}}\rangle$, and $|e_{Vx_{2}}\rangle$ are the unnormalized states of Eve’s probes. □

When Charlie receives the qubits, he selects the SIFT operation or CTRL operation. Subsequently, Bob performs ${\bar{U}}_{F}$ on the state.

Charlie selects the SIFT operation. The global state is collapsed into one of four states: $|Hx_{1}\rangle |e_{Hx_{1}}\rangle$, $|Hx_{2}\rangle |e_{Hx_{2}}\rangle$, $|Vx_{1}\rangle |e_{Vx_{1}}\rangle$, and $|Vx_{2}\rangle |e_{Vx_{2}}\rangle$. If Bob wants no error to be introduced, then after ${\bar{U}}_{F}$, there are

(15) $$\begin{array}{c} {\bar{U}}_{F}(|Hx_{1}\rangle |e_{Hx_{1}}\rangle )=|Hx_{1}\rangle |f_{Hx_{1}}\rangle , \end{array}$$

(16) $$\begin{array}{c} {\bar{U}}_{F}(|Hx_{2}\rangle |e_{Hx_{2}}\rangle )=|Hx_{2}\rangle |f_{Hx_{2}}\rangle , \end{array}$$

(17) $$\begin{array}{c} {\bar{U}}_{F}(|Vx_{1}\rangle |e_{Vx_{1}}\rangle )=|Vx_{1}\rangle |f_{Vx_{1}}\rangle , \end{array}$$

(18) $$\begin{array}{c} {\bar{U}}_{F}(|Vx_{2}\rangle |e_{Vx_{2}}\rangle )=|Vx_{2}\rangle |f_{Vx_{2}}\rangle . \end{array}$$

Charlie selects the CTRL operation. The global state should be unchanged:

(19) $$\begin{array}{c} {\bar{U}}_{F}(Hx_{1}\rangle |e_{Hx_{1}}\rangle +|Hx_{2}\rangle |e_{Hx_{2}}\rangle +|Vx_{1}\rangle |e_{Vx_{1}}\rangle +|Vx_{2}\rangle |e_{Vx_{2}}\rangle )\\ =|Hx_{1}\rangle |f_{Hx_{1}}\rangle +|Hx_{2}\rangle |f_{Hx_{2}}\rangle +|Vx_{1}\rangle |f_{Vx_{1}}\rangle +|Vx_{2}\rangle |f_{Vx_{2}}\rangle , \end{array}$$

According to the protocol, if there is no error introduced, then from Equation (19), it must hold that

(20) $$\begin{array}{c} |f_{Hx_{1}}\rangle =|f_{Hx_{2}}\rangle =|f_{Vx_{1}}\rangle =|f_{Vx_{2}}\rangle =|f\rangle . \end{array}$$

According to Equation (20), Equations (15)–(18) can be deduced:

(21) $$\begin{array}{c} {\bar{U}}_{F}(|Hx_{1}\rangle |e_{Hx_{1}}\rangle )=|Hx_{1}\rangle |f_{Hx_{1}}\rangle =|Hx_{1}\rangle |f\rangle , \end{array}$$

(22) $$\begin{array}{c} {\bar{U}}_{F}(|Hx_{2}\rangle |e_{Hx_{2}}\rangle )=|Hx_{2}\rangle |f_{Hx_{2}}\rangle =|Hx_{2}\rangle |f\rangle , \end{array}$$

(23) $$\begin{array}{c} {\bar{U}}_{F}(|Vx_{1}\rangle |e_{Vx_{1}}\rangle )=|Vx_{1}\rangle |f_{Vx_{1}}\rangle =|Vx_{1}\rangle |f\rangle , \end{array}$$

(24) $$\begin{array}{c} {\bar{U}}_{F}(|Vx_{2}\rangle |e_{Vx_{2}}\rangle )=|Vx_{2}\rangle |f_{Vx_{2}}\rangle =|Vx_{2}\rangle |f\rangle . \end{array}$$

To avoid introducing errors during Alice’s eavesdropping detection, the final state of Bob’s probes should be independent of Charlie’s measurement results. Theorem 1 has been proven by the above methods.

## 4. Discussion and Conclusions

Now, we discuss the differences between our proposed protocol and the existing SQSS protocol. Obviously, compared with the previous SQSS protocol, the proposed protocol features the use of hyper-entangled states in two DoFs, which improves the communication capacity of the protocol. The qubit efficiency can be defined as $\eta =\frac{b}{q}$ [38], where *b* denotes the total number of shared classical bits and *q* denotes the total number of prepared qubits in the protocol. Specific comparisons are given in Table 4.

The application research on multi-DoF quantum information has just started. The multi-DoF approach may give people more colorful content which differs from manipulating a single DoF. In this paper, by using hyper-entangled states as quantum transmission carriers, we proposed a semi-quantum secret-sharing protocol with high efficiency which solves the problem of only the cooperation of different participants being able to recover the key. The analysis results show that the protocol can detect well-known attacks, such as an intercept-resend attack, measure-resend attack, and entangle-measure attack, and thus the security is asymptotically secure in theory. More in-depth security analysis is needed in practice. Applying hyper-entangled states in quantum communication can improve the channel capacity and communication security, serve practical quantum communication networks, and promote the practical process of semi-quantum cryptography communication.

## Figures and Tables

**Table 1:** Explanations of acronyms and symbols.

Case	Bob’s Operation
QSS	Quantum secret sharing
SQSS	Semi-quantum secret sharing
DoF	Degree of freedom
DoFs	Degrees of freedom
QKD	Quantum key distribution
SQKD	Semi-quantum key distribution
SIFT	Measure and prepare the qubits with Z basis
CTRL	Reflect the qubits without disturbance
X_P(X_S)	X basis under polarization DoF (spatial mode DoF)
Z_P(Z_S)	Z basis under polarization DoF (spatial mode DoF)
K_A	Alice’s secret bit
K_B	Bob’s secret bit
K_C	Charlie’s secret bit
η	Qubit efficency

**Table 2:** Participants’ operations on the qubits in each position.

Case	Bob’s Operation	Charlie’s Operation	Alice’s Operation	Usage
1	SIFT	SIFT	Measure the qubits *B* and *C* with $Z_{P}⊗Z_{S}$	Generate raw key, check for eavesdropping
2	SIFT	CTRL	Measure the qubits *B* with $Z_{P}⊗Z_{S}$ and measure the qubits *C* with $X_{P}⊗X_{S}$	Check for eavesdropping
3	CTRL	SIFT	Measure the qubits *B* with $X_{P}⊗X_{S}$ and Measure the qubits *C* with $Z_{P}⊗Z_{S}$	Check for eavesdropping
4	CTRL	CTRL	Measure the qubits *B* and *C* with $X_{P}⊗X_{S}$	Check for eavesdropping

**Table 3:** The shared secret information between two participants from Alice.

Secret Information 1 and 2	Bob’s Results	Charlie’s Results	Alice’s Results
0 and 0	${|0\rangle }_{P}^{}⊗{|0\rangle }_{S}^{}$	${|0\rangle }_{P}^{}⊗{|0\rangle }_{S}^{}$	${|00\rangle }_{P}^{}⊗{|00\rangle }_{S}^{}$
0 and 1	${|0\rangle }_{P}^{}⊗{|0\rangle }_{S}^{}$	${|0\rangle }_{P}^{}⊗{|1\rangle }_{S}^{}$	${|00\rangle }_{P}^{}⊗{|01\rangle }_{S}^{}$
1 and 0	${|0\rangle }_{P}^{}⊗{|0\rangle }_{S}^{}$	${|1\rangle }_{P}^{}⊗{|0\rangle }_{S}^{}$	${|01\rangle }_{P}^{}⊗{|00\rangle }_{S}^{}$
1 and 1	${|0\rangle }_{P}^{}⊗{|0\rangle }_{S}^{}$	${|1\rangle }_{P}^{}⊗{|1\rangle }_{S}^{}$	${|01\rangle }_{P}^{}⊗{|01\rangle }_{S}^{}$
0 and 1	${|0\rangle }_{P}^{}⊗{|1\rangle }_{S}^{}$	${|0\rangle }_{P}^{}⊗{|0\rangle }_{S}^{}$	${|00\rangle }_{P}^{}⊗{|10\rangle }_{S}^{}$
0 and 0	${|0\rangle }_{P}^{}⊗{|1\rangle }_{S}^{}$	${|0\rangle }_{P}^{}⊗{|1\rangle }_{S}^{}$	${|00\rangle }_{P}^{}⊗{|11\rangle }_{S}^{}$
1 and 1	${|0\rangle }_{P}^{}⊗{|1\rangle }_{S}^{}$	${|1\rangle }_{P}^{}⊗{|0\rangle }_{S}^{}$	${|01\rangle }_{P}^{}⊗{|10\rangle }_{S}^{}$
1 and 0	${|0\rangle }_{P}^{}⊗{|1\rangle }_{S}^{}$	${|1\rangle }_{P}^{}⊗{|1\rangle }_{S}^{}$	${|01\rangle }_{P}^{}⊗{|11\rangle }_{S}^{}$
1 and 0	${|1\rangle }_{P}^{}⊗{|0\rangle }_{S}^{}$	${|0\rangle }_{P}^{}⊗{|0\rangle }_{S}^{}$	${|10\rangle }_{P}^{}⊗{|00\rangle }_{S}^{}$
1 and 1	${|1\rangle }_{P}^{}⊗{|0\rangle }_{S}^{}$	${|0\rangle }_{P}^{}⊗{|1\rangle }_{S}^{}$	${|10\rangle }_{P}^{}⊗{|01\rangle }_{S}^{}$
0 and 0	${|1\rangle }_{P}^{}⊗{|0\rangle }_{S}^{}$	${|1\rangle }_{P}^{}⊗{|0\rangle }_{S}^{}$	${|11\rangle }_{P}^{}⊗{|00\rangle }_{S}^{}$
0 and 1	${|1\rangle }_{P}^{}⊗{|0\rangle }_{S}^{}$	${|1\rangle }_{P}^{}⊗{|1\rangle }_{S}^{}$	${|11\rangle }_{P}^{}⊗{|01\rangle }_{S}^{}$
1 and 1	${|1\rangle }_{P}^{}⊗{|1\rangle }_{S}^{}$	${|0\rangle }_{P}^{}⊗{|0\rangle }_{S}^{}$	${|10\rangle }_{P}^{}⊗{|10\rangle }_{S}^{}$
1 and 0	${|1\rangle }_{P}^{}⊗{|1\rangle }_{S}^{}$	${|0\rangle }_{P}^{}⊗{|1\rangle }_{S}^{}$	${|10\rangle }_{P}^{}⊗{|11\rangle }_{S}^{}$
0 and 1	${|1\rangle }_{P}^{}⊗{|1\rangle }_{S}^{}$	${|1\rangle }_{P}^{}⊗{|0\rangle }_{S}^{}$	${|11\rangle }_{P}^{}⊗{|10\rangle }_{S}^{}$
0 and 0	${|1\rangle }_{P}^{}⊗{|1\rangle }_{S}^{}$	${|1\rangle }_{P}^{}⊗{|1\rangle }_{S}^{}$	${|11\rangle }_{P}^{}⊗{|11\rangle }_{S}^{}$

**Table 4:** SQSS protocol comparison.

Reference	Quantum Source	Measurement	Decoy Photons	DoF	Sharing Message	Qubit Effigency
[27]	GHZ-type states	Single qubit measurement, Bell measurement, three-qubit joint measurement	No	1	Unspecific	$\frac{1}{8}$
[28]	Two-qubit product states	Single qubit measurement	No	1	Unspecific	$\frac{1}{4}$
[29]	Two entangled states	Single qubit measurement, two-particle measurement, three-particle measurement	No	1	Specific	$\frac{1}{4}$
[30]	Bell states	Single qubit measurement, Bell measurement	No	1	Unspecific	-
[31]	Two-qubit product states	Single qubit measurement	No	1	Unspecific	$\frac{1}{4}$
[32]	Two-qubit product states	-	No	1	Unspecific	$\frac{1}{8}$
[33]	W states	Single qubit measurement, Bell measurement	No	1	Unspecific	$\frac{1}{8}$
[34]	Bell states	Single qubit measurement, Bell measurement	Yes	1	Specific	<$\frac{1}{2}$
[35]	Product states	Single qubit measurement	No	1	Unspecific	$\frac{1}{4}$
Proposed protocol	Two-qubit product hyper-entangled states	Single hyper-entangled qubit measurement	No	2	Unspecific	$\frac{1}{2}$

## Data Availability

Not applicable.
